# Investigating the Mechanisms of Bisdemethoxycurcumin in Ulcerative Colitis: Network Pharmacology and Experimental Verification

**DOI:** 10.3390/molecules28010068

**Published:** 2022-12-21

**Authors:** Huihuan Wu, Sha Tu, Zewei Zhuo, Rui Jiang, Ruijie Zeng, Qi Yang, Qizhou Lian, Weihong Sha, Hao Chen

**Affiliations:** 1 Department of Gastroenterology, Guangdong Provincial People’s Hospital, Guangdong Academy of Medical Sciences, Guangzhou 510080, China; 2 School of Medicine, South China University of Technology, Guangzhou 510006, China; 3 Department of Medicine, Queen Mary Hospital, Hong Kong SAR 999077, China

**Keywords:** bisdemethoxycurcumin, ulcerative colitis, network pharmacology, PI3k/AKT pathway, MAPK pathway

## Abstract

Ulcerative colitis is a chronic inflammatory bowel disorder that is hard to cure once diagnosed. Bisdemethoxycurcumin has shown positive effects on inflammatory diseases. However, the underlying bioactive interaction between bisdemethoxycurcumin and ulcerative colitis is unclear. The objective of this study was to determine the core target and potential mechanism of action of bisdemethoxycurcumin as a therapy for ulcerative colitis. The public databases were used to identify potential targets for bisdemethoxycurcumin and ulcerative colitis. To investigate the potential mechanisms, the protein-protein interaction network, gene ontology analysis, and Kyoto encyclopedia of genes and genomes analysis have been carried out. Subsequently, experimental verification was conducted to confirm the findings. A total of 132 intersecting genes of bisdemethoxycurcumin, as well as ulcerative coli-tis-related targets, were obtained. SRC, EGFR, AKT1, and PIK3R1 were the targets of highest potential, and the PI3K/Akt and MAPK pathways may be essential for the treatment of ulcerative colitis by bisdemethoxycurcumin. Molecular docking demonstrated that bisdemethoxycurcumin combined well with SRC, EGFR, PIK3R1, and AKT1. Moreover, the in vitro experiments suggested that bisdemethoxycurcumin might reduce LPS-induced pro-inflammatory cytokines levels in RAW264.7 cells by suppressing PI3K/Akt and MAPK pathways. Our study provided a comprehensive overview of the potential targets and molecular mechanism of bisdemethoxycurcumin against ulcerative colitis. Furthermore, it also provided a theoretical basis for the clinical treatment of ulcerative colitis, as well as compelling evidence for further study on the mechanism of bisdemethoxycurcumin in the treatment of ulcerative colitis.

## 1. Introduction

Ulcerative colitis (UC) is one of the two major types of inflammatory bowel disease (IBD) that causes recurring and remitting inflammation of the digestive tract [1]. During the past few decades, the incidence and prevalence of UC have been continuously increasing [1]. Furthermore, patients who have suffered from UC for a long time are more likely to develop related cancers, which can negatively impact their physical and mental health [2]. Currently, 5-aminosalicylic acid drugs, steroids, immunosuppressants, and biological drugs are the main treatments for UC [3]. Despite the wide range of therapeutic options available, there are still limitations in terms of efficacy and safety [4]. Thus, it is critical to investigate natural ingredients and their derivative products as therapeutic and management aids for UC.

Curcuminoids, polyphenol coloring compounds, were extracted from *Curcuma longa.* The main components of curcuminoids found in *Curcuma longa* are curcumin, demethoxycurcumin, and bisdemethoxycurcumin (BDMC) [5]. Compared with curcumin, BDMC exhibits superior stability under biological conditions and enhanced cellular uptake [6,7]. According to previous studies, BDMC possesses antioxidant and anti-inflammatory properties [6,8,9,10]. However, there are no studies investigating the therapeutic effects, target genes, and underlying mechanisms of BDMC in the treatment of UC. Therefore, the research of potential target genes and pharmacological mechanisms of BDMC may provide a new approach for the treatment of UC.

Network pharmacology is a brand-new field that combines high throughput histology, bioinformatics, and system biology, which has been extensively applied to investigate the mechanism underlying the curative action of traditional Chinese medicine [11]. In this research, network pharmacology was used to investigate the pharmacologic mechanism of BDMC in UC in conjunction with docking analysis and in vitro verification.

## 2. Results

### 2.1. Targets of BDMC and UC-Related Targets

**Figure 1:**
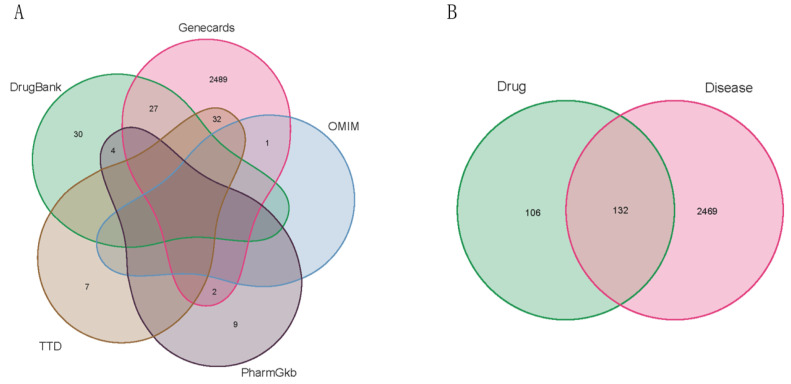
(**A**) Venn diagrams showing UC targets obtained from five databases, (**B**) the intersection of identified target genes of BDMC and UC.

The MOL2 structure of BDMC was downloaded from the TCMSP (Molecule ID: MOL000940). A total of 240 targets of BDMC were obtained from the PharmMapper server (Supplementary Table S1). Furthermore, target genes associated with UC were retrieved from the DrugBank, GeneCards (Relevance score ≥ 1, Supplementary Table S2), OMIM, PharmGkb, TTD databases, which included 180 genes in DrugBank, 2551 in GeneCards, 1 in OMIM, 15 in PharmGkb, and 42 in TTD using the keyword “Ulcerative Colitis. There were 132 overlapping recognization targets between 240 BDMC-related targets and 2601 UC-related targets obtained by intersection (Figure 1A and Supplementary Table S3), which were designated as hotspot targets for further investigation. (Figure 1B and Supplementary Table S4)

### 2.2. Construction of BDMC-UC-Related PPI Network

**Figure 2:**
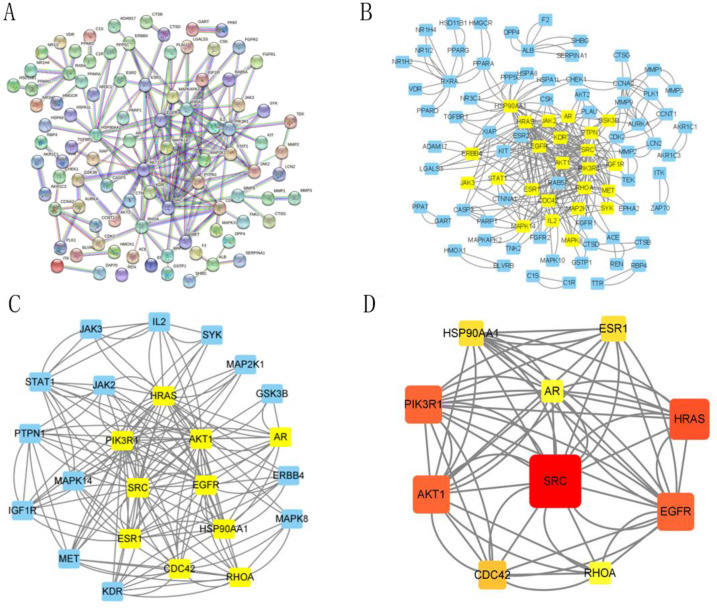
PPI network of BDMC-UC. (**A**) The interactive PPI network derived from STRING database. (**B**) PPI network obtained from STRING database to Cytoscape3.8.0. (**C**) PPI network of more important proteins extracted from (**B**) by filtering six parameters: BC, CC, DC, EC, NC, and LAC. (**D**) Core PPI network of key proteins extracted from (**C**). Higher degree values indicate larger node sizes, red represents a higher degree, and yellow represents a lower degree.

The string database created a PPI network with 169 edges and 132 nodes, where edges represent protein-protein and nodes represent proteins (Figure 2A). The PPI data was then acquired into Cytoscape3.8.0 to create a brand-new PPI network with 132 nodes and 169 edges to visualize and assess the protein-protein interactions (Figure 2B). Following that, the CytoNCA plugin in Cytoscape3.8.0 was used to mine the core goals. Based on the relevant median values, the following were the selection process: BC greater than 0, CC greater than 0.050304097, DC greater than 4, EC greater than 0.024781074, NC greater than 0, and LAC greater than 0. In Figure 2B, gene targets meeting the inclusion criteria were kept and highlighted in yellow. As shown in Figure 2C, we created a new PPI network with 75 nodes and 307 edges based on gene targets that met the inclusion criteria. Similarly, only targets meeting the inclusion criteria (BC > 7.8865800865, CC > 0.56097561, DC >12, EC > 0.179094382, NC > 5.766233766, and LAC > 5.1666666665) were kept and highlighted in yellow (Figure 2C). Eventually, we screened out a key PPI network with 18 nodes and 57 edges (Figure 2D). SRC (*n* = 18), EGFR (*n* = 16), AKT1 (*n* = 14), PIK3R1 (*n* = 14), AR (*n* = 12), HSP90AA1 (*n* = 12), CDC42 (*n* = 10), HRAS (*n* = 10), ESR1 (*n* = 10), and RHOA (*n* = 8), which may be extremely important in the anti-UC of BDMC, were the highest degree-valued target nodes.

### 2.3. Functional Enrichment Analysis

**Figure 3:**
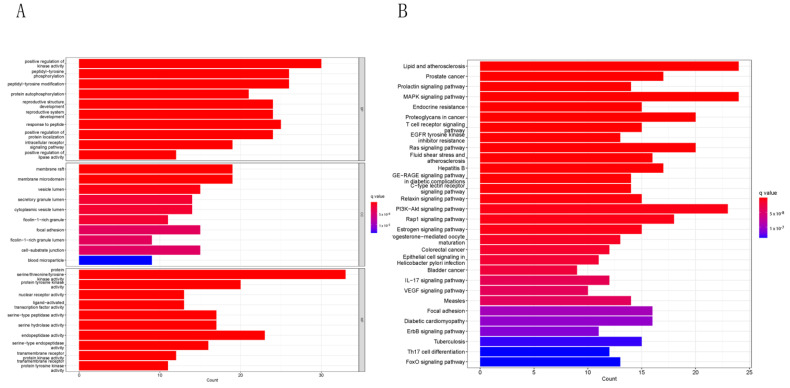
(**A**) The bar plot diagram of GO analysis of BDMC-UC, including the top 10 important enrichment terms of BP, CC, and MF. Redder means lower q value. (**B**) KEGG enrichment analysis for the major target of BDMC (top 30). Redder means lower q value.

GO and KEGG pathway analyses were performed to investigate the biological functions and pathways linked to the possible anti-UC genes of BDMC. The top 10 significant enrichment terms of biological processes (BP), cellular components (CC), and molecular function (MF) were chosen for presentation (Figure 3A). As suggested from the results, the potential anti-UC genes of BDMC were mainly enriched in protein serine/threonine/tyrosine kinase activity, positive regulation of kinase activity, peptidyl-tyrosine phosphorylation, and peptidyl-tyrosine modification. For the KEGG pathway analysis, lipid and atherosclerosis, the MAPK pathway, and the PI3K/Akt pathway were most vastly associated with the potential anti-UC genes of BDMC (Figure 3B), suggesting that BDMC may exert its therapeutic value for UC by modulating the above signaling pathways.

### 2.4. BDMC-Target-Pathway Network

**Figure 4:**
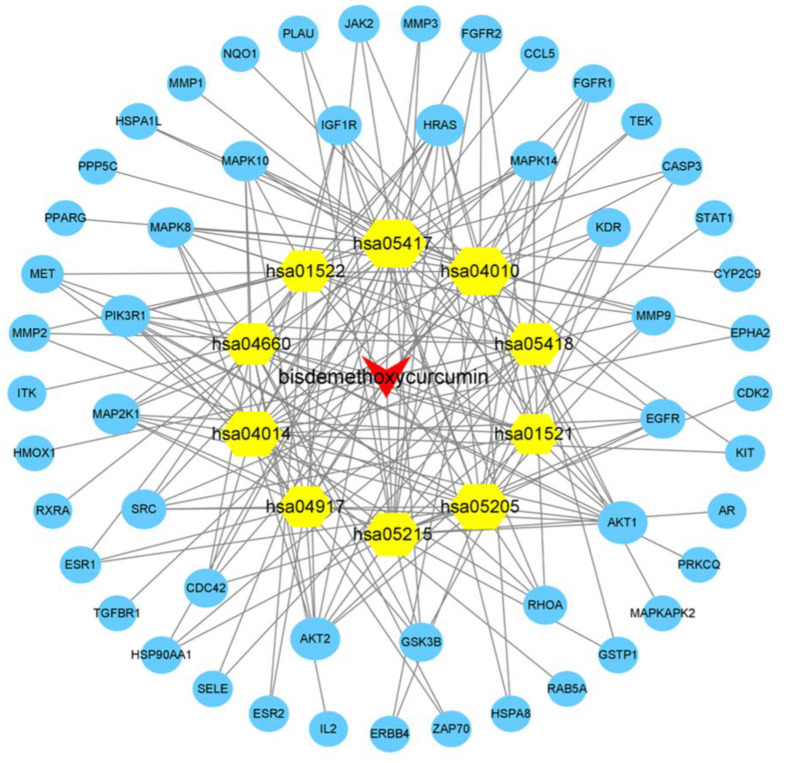
The construction BDMC-target-pathway network. The red inverted triangles represent BDMC, the blue ellipses represent target genes, and the yellow hexagons represent the top 10 KEGG pathways of BDMC treating UC. The gray lines represent their interaction.

A BDMC-target-pathway network was established to demonstrate the relationship between BDMC, common targets, and their correlated pathways. As shown in Figure 4, the BDMC-target-pathway interaction network works with 64 nodes and 188 edges.

### 2.5. Verification of BMDC-Target Interaction Interaction

**Figure 5:**
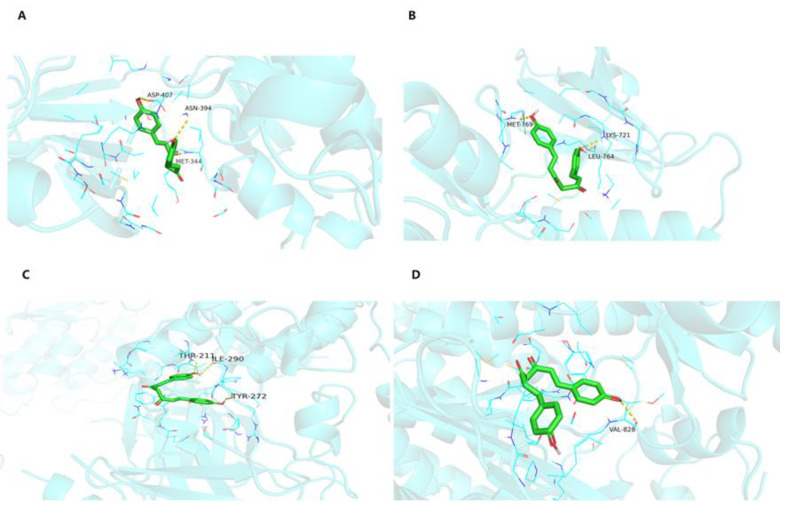
Molecular docking models of BDMC binding to major hub targets. (**A**) SRC with BDMC. (**B**) EGFR with BDMC. (**C**) AKT1 with BDMC. (**D**) PI3KR1 with BDMC. The yellow dashed lines represent hydrogen, whereas the red lines represent π-π interactions.

Molecular docking was used to investigate the interaction between BDMC and the four targets (SRC, EGFR, AKT1, and PI3KR1 in the PPI network). The binding energy is less than 5.0 kcal/mol, indicating that the confirmations have good interactions [12]. As shown in Table 1, there are good binding and reliable interactions between BDMC and major targets. The conformations of BDMC and the top three targets were shown in Figure 5.

### 2.6. Cytotoxic Effect of BDMC in RAW264.7 Cell

**Figure 6:**
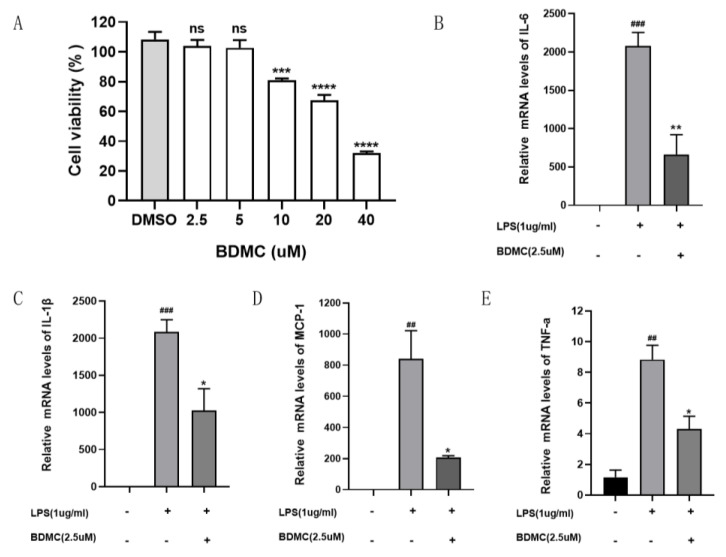
(**A**) BDMC as indicated. Cell viability was determined by CCK8 assays. Data are presented as the mean ± SEM. *** *p* < 0.001, **** *p* < 0.0001 vs. vehicle control (DMSO). BDMC decreases the levels of proinflammatory factors (**B**–**E**). RAW264.7 cells were pre-treated for 1 h with 2.5 uM concentrations of bisdemethoxycurcumin and incubated with or without LPS (1 μg/mL) for 24 h. The expressions of IL−6 (**B**), IL−1β (**C**), MCP−1 (**D**), and TNF−a mRNA levels were examined by real-time PCR. Data are presented as the mean ± SEM. ^##^ *p* < 0.01; ^###^ *p* < 0.001 vs. control group; * *p* < 0.05; ** *p* < 0.01; *** *p* < 0.001; **** *p* <0.0001 vs. LPS group.

The CCK8 assay was utilized to demonstrate the impact of BDMC on the viability of RAW264.7 cells. As shown in Figure 6A, BDMC at concentrations of 2.5 μM and 5 μM has no cytotoxic effect on RAW264.7 cells. Thus, further studies were carried out with 2.5 μM of BDMC.

### 2.7. BDMC’s Effect on Inflammatory Cytokines in LPS-Induced RAW 264.7 Cells

To confirm the anti-inflammatory effects of BDMC, the expression of IL-6, IL-1β, TNF-a, and MCP-1 in LPS-induced RAW 264.7 cells were detected by real-time PCR. As presented in Figure 6B–E, LPS notably increased the levels of IL-6 (Figure 6B), IL-1β (Figure 6C), MCP-1 (Figure 6D), and TNF-a (Figure 6E) compared to the control group, and pre-treatment with BDMC significantly reduced the expression of IL-6, IL-1β, TNF-a, and MCP-1.

### 2.8. BDMC Suppressed the Protein Levels of MAPK and PI3K/Akt Pathways in LPS-Induced RAW 264.7 Cells

**Figure 7:**
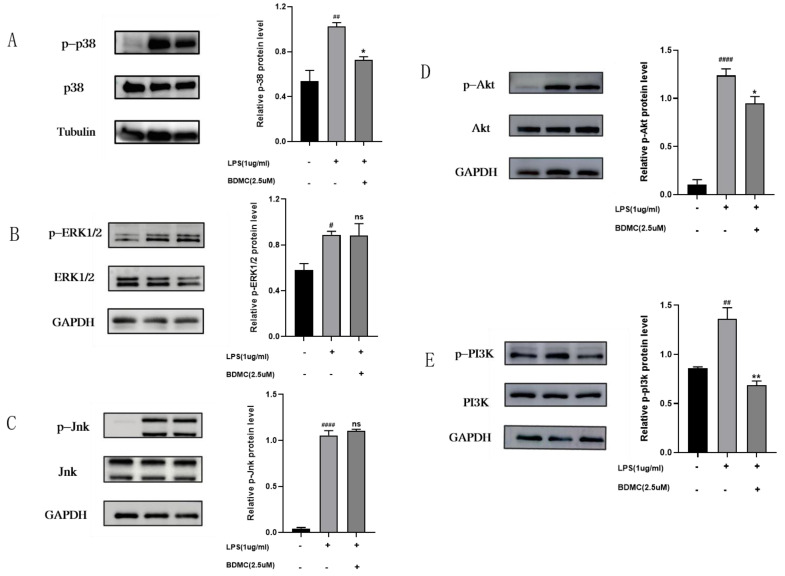
The protein expression levels of p-p38, p38, p-ERK1/2, ERK1/2, p-Jnk, Jnk, p-Akt, Akt, p-PI3k, PI3k, GAPDH, and tubulin in LPS-induced RAW264.7 after giving BMDC. (**A**) The protein expression levels of p-p38, p-38. (**B**) The protein expression levels of p-ERK1/2, ERK1/2. (**C**) The protein expression levels of p-Jnk, Jnk. (**D**) The protein expression levels of p-Akt, Akt. (**E**) The protein expression levels of p-PI3K, PI3K.Data are presented as the mean ± SEM. ^#^ *p* < 0.05, ^##^ *p* < 0.01; ^####^ *p* < 0.001 vs. control group; * *p* < 0.05; ** *p* < 0.01 vs. LPS group. ns mean non-significant vs. LPS group.

The previous results have revealed that lipid and atherosclerosis, the MAPK pathway, and the PI3K-Akt pathway were most greatly linked to the potential anti-UC genes of BDMC. Thus, the phosphorylation of key protein kinases of the MAPK and PI3K-Akt pathways were detected to explore the underlying mechanism of BDMC on the treatment of UC. As presented in (Figure 7A–E), compared to the control group, the phosphorylation levels of Akt, PI3k, p38, Jnk, and ERK1/2 were higher after being stimulated by LPS in RAW264.7 cells. Moreover, pretreatment with BDMC remarkably decreased the levels of p-Akt, p-PI3K, and p-p38 protein expression. However, the administration of BDMC did not affect the phosphorylation of ERK1/2 and Jnk.

## 3. Discussion

In this study, we predicted the mechanism of BDMC in the treatment of UC, based on network pharmacology, and verified those results with in vitro experiments. The results of network pharmacology demonstrated that the underlying mechanism for the BDMC in the treatment of UC is complex, involving multiple protein targets and pathways; SRC, EGFR, AKT1, and PIK3R1 were the top four core potential targets. The molecular docking results showed that BDMC and AKT1 exhibited the best binding interaction. According to the GO and KEGG analyses, the most significant pathways which were involved in the treatment of UC by BDMC were lipid and atherosclerosis, the MAPK pathway, and the PI3K-Akt pathway. Our study is the first study to elucidate the underlying mechanism of BDMC in the treatment of UC using network pharmacology, which provides a theoretical starting point for the clinical treatment of UC.

Our study results revealed that multiple genetic and molecular interactions between BDMC and UC via PPI network analysis and drug -target-disease pathway analysis. After a PPI topological screening, SRC, EGFR, AKT1, and PIK3R1 were the top four core targets. It implies that BDMC might be crucial in treating UC by affecting these targets. Furthermore, we performed molecular docking to further verify the candidate core targets. Based on binding potency and hydrogen bonding results (Table 1), the bond between BDMC and AKT1 was the most stable. Serine/threonine protein kinases Akt1, Akt2, and Akt3 are members of the Akt family; AKT protein kinase plays key roles in a number of cellular processes, such as cell proliferation, apoptosis, transcription, and cell migration [13,14]. As reported, during the progression of UC, the PI3K/AKT pathway is extremely crucial in the regulation of the inflammatory reaction. In UC, unusual PI3K/Akt pathway activation can have a major impact on the expression and secretion of proinflammatory cytokines, which are crucial for the progression of UC [15,16]. The results of the KEGG analysis demonstrated that the PI3K/Akt pathways were significantly associated with the potential anti-UC genes of BDMC. Thus, we hypothesized that BDMC could reduce the levels of proinflammatory cytokines through regulating the PI3K/Akt pathway. Subsequent in vitro experimentation results verified that BDMC may reduce the release of the proinflammatory cytokines. Furthermore, BDMC inhibited the PI3K/Akt pathway activation; Therefore we speculated that BDMC may alleviated UC by reducing the release of the proinflammatory cytokines via inhibiting PI3K/Akt pathway activation. The result is consistent with the network pharmacology approach. In spite of this, this result has still not been reported in previous trials of BDMC treatment for UC. However, in other tumor models related studies, BDMC has been reported to cause inhibited proliferation, induce apoptosis, and suppress cell invasion and migration by suppressing the PI3K/Akt pathway activation [17,18,19]. This finding provided us with insight into whether BDMC is effective in treating UC via a similar mechanism.

In addition to the PI3K/Akt pathway, the KEGG analysis also found that target genes were similarly enriched in the MAPK pathway. Several studies have shown that the MAPK signaling pathway is closely correlated with UC. Zhao X et al., demonstrated that the level of P-P38 MAPK was enhanced in UC patients versus control samples [20]. Fan H et al., found that p-p38 MAPK activation in DSS-induced colitis is related with elevated TNF-a and MPO levels [21].

At the same time, our experiment in vitro showed that the protein expression of p-p38 MAPK, p-ERK1/2, and p-JNK were detected, and the expression of p-p38 MAPK was obviously reduced in LPS-induced RAW264.7 cells, while that has no effects on p- p-ERK1/2 and p-JNK. Therefore, we speculated that BDMC may alleviate UC via inhibiting the p-p38 MAPK signaling pathway activation, instead of inhibiting p-ERK1/2 and p-JNK signaling pathway activation. Taken together, our research shows that the PI3K/Akt pathway and p-p38 MAPK pathway may be crucial in the treatment of UC with BDMC.

However, there are several limitations to our study. First of all, due to incomplete database information, some critical target genes may be missing. Secondly, various signaling pathways in which BDMC is operating on UC were speculated employing the network pharmacological strategies; howeverthe contribution of each pathway was not identified. Finally, our current findings only provide the direction of future research, whereas the specific mechanisms still need to be carefully investigated in subsequent studies.

## 4. Materials and Methods

### 4.1. Targets Prediction for Bisdemethoxycurcumin

The MOL2 structure of BDMC was provided from the Traditional Chinese Medicine Systems Pharmacology Database and Analysis Platform (TCMSP), which was inputted into the Pharm Mapper server (http://www.lilab-ecust.cn/pharmmapper/, accessed on 1 May 2022) to obtain the targets of the pharmacophore model and 299 targets of BDMC were screened by Norm Fit ≥0.3.

### 4.2. Collection of Ulcerative Colitis-Related Targets

Therapeutic targets of ulcerative colitis were offered by five public databases with the keywords “ulcerative colitis”: Gene Cards (https://www.genecards.org/, accessed on 1 May 2022), Drug Bank (https://go.drugbank.com/, accessed on 1 May 2022), Pharm GKB (https://www.pharmgkb.org/, accessed on 1 May 2022), Therapeutic Target Database (TTD, http://db.idrblab.net/ttd/, accessed on 1 May 2022), and Online Mendelian Inheritance in Man (OMIM, https://omim.org/, accessed on 1 May 2022). All targets were filtered as “homo sapiens”.

### 4.3. Targets for BDMC in the Therapy of UC

The candidate targets of BDMC and UC-related targets were integrated together and delineated with the Venn diagram plotted by the R language Venn Diagram package for intuitive vision.

### 4.4. Conducting Protein-Protein Interaction (PPI) Network

The BDMC and UC molecular targets were obtained to construct a PPI network by using the STRING database (https://string-db.org/, accessed on 2 May 2022), with the organism parameter set to homo sapiens, the required minimal interaction ratio of 0.95, and hiding individual target protein nodes, as well as other basic settings left at the default value. The data obtained from the String database were inputted into Cyto-scape3.8.0 to visualize the PPI network. Six topological node metrics were calculated using the CytoNCA plugin in Cytoscape3.8.0, which include network centrality (NC), betweenness centrality (BC), closeness centrality (CC), eigenvector centrality (EC), degree centrality (DC), and local average connectivity (LAC). These parameters may all be used to analyze the characteristics of nodes in an interactive network in-depth. Metrics with higher quantitative values indicate that nodes in a network are more important. To create a brand-new PPI network for further study, target nodes were kept only if all six metrics exceeded their respective medians. The network was handled as a non-directed graph and node degree distribution diagrams were created using the Network Analyzer Cytoscape plugin. Six parameters were also determined for the new network and only nodes having all six parameters greater than their respective median values were kept in place for establishing.

### 4.5. Function and Pathway Enrichment Analysis

To investigate the roles of potential anti-UC target genes of BDMC, the gene ontology (GO) analysis and the Kyoto encyclopedia of genes and genomes (KEGG) analysis were employed. Only routes and functional words with q values of less than 0.05 were statistically meaningful and maintained.

### 4.6. Network Construction

The software Cytoscape3.8.0 was employed to create a BDMC-target-pathway network and analyze the relationship between compounds, targets, as well as ulcerative colitis.

### 4.7. Molecular Docking

The 2D structure of BDMC was downloaded from PubChem (https://pubchem.ncbi.nlm.nih.gov/, accessed on 3 May 2022), then it was imported into Chem Draw 3D to minimize energy through the MM 2 module, and choose the best conformation with the lowest energy, and save it as a mol 2 file. The Protein Data Bank (PDB) (http://www.rcsb.org/, accessed on 3 May 2022) was used to obtain the crystal structures of core protein targets. The BDMC and core protein targets were converted to the PDBQT format using the AutoDockTools software(https://autodock.scripps.edu/, accessed on 9 May 2022). The AutoDockVina was applied to undertake molecular docking and determine the binding energy. The docking outcome of BDMC and the core protein targets were visualized using the PyMOL software (https://pymol.org/2/, accessed on 9 May 2022).

### 4.8. Cytotoxicity Test

The Cell Counting Kit-8 (Guangzhou Bin Peng Biotechnology Co., Ltd., Guangzhou, China) was used to evaluate the effects of BDMC (Aladdin Biochemical Technology Company, Beijing, China) in RAW264.7 cell lines. RAW264.7 cells were seeded at a density of 5 × 104 cells per in-96-wall panel. After, 12 h, the medium was changed to brand-new 1640 (Gibco, New York, NY, USA) that contained various concentrations of BDMC (2.5, 5, 10, 20, and 40 μM). After 48 h, the culture was stopped, and 10 μL of CCK8 reagent was added, which was then incubated for 1 h at 37 °C. The absorbance at 450 nm was measured using a microplate reader.

### 4.9. Quantitative Reverse Transcription-PCR

RAW264.7 cells were planted at a density of 5 × 10^6^ cells per well in 6-well panels. After being incubated for 12 h, the cells were either pre-treated for 1 h with BDMC and an LPS treatment with 1 μg/mL or left untreated for 24 h. Total RNA was extracted from cells using the Total RNA Isolation Kit (Omega, New York, GA, USA). Reverse transcription was carried out using the Prime Script TM RT-PCR kit from Takara, Otsu, Japan, and the Biorad CFX Connect (Biorad, Hercules, CA, USA) was used for qRT-PCR, as directed by the manufacturer. This research used the following primers: interleukin-6 (IL-6): 5′-CTGCAAGAGACTTCCATCCAG-3′ (forward) and 5′-AGTGGTATAGA-CAGGTCTGTTGG-3′ (reverse); monocyte chemoattractant protein-1 (MCP-1): 5′-CTGTGCTGACCCCAAGAAGG-3′ (forward) and 5′-AGGTGGTTGTGGAAAAGGTAGTG-3′ (reverse); interleukin-1β(IL-1β): 5′-ATGCCACCTTTTGACAGTGATG-3′ (forward) and 5′-TGATGTGCTGCTGCGAGATT-3′ (reverse); tumor necrosis factor alpha (TNF-a): 5′-CGACGTGGAACTGGCAAGAA-3′ (forward) and 5′-GGACCGATCACCCCGAAG-3′ (reverse); and GAPDH: 5′-AGGTCGGTGTGAACGGATTTG-3′ and 5′-TGTAGACCATGTAGTTGAGGTCA-3′ (reverse). The 2−∆∆Ct method was used to calculated relative expressions of mRNAs.

### 4.10. Western Blot Analysis

RAW264.7 cells were planted in 6-well plates at a density of 5 × 10^6^ cells per well, pre-treated for 1 h with BDMC and LPS treatment with 1 μg/mL, or left untreated for 24 h. The Western blot was performed exactly as previously described [22]. Primary antibodies of GAPDH, Tubulin, p-Akt, Akt, p-pI3K, pI3K, p-JNK, JNK, p-ERK1/2, ERK1/2, p-p38MAPK, and p38MAPK were purchased from Cell Signaling Technology (Danvers, MA, USA). The findings were presented as means ± S.E.M. GraphPad Prism 8.0.2 was used to analyze the results, and the data comparison of multiple groups was analyzed by Ordinary one-way ANOVA. A *p* value of less than 0.05 was considered statistically significant.

## 5. Conclusions

We looked into the potential mechanisms of BDMC using network pharmacology, molecular docking, and in vitro verification. We believe that this research has huge value in terms of providing a theoretical basis for the clinical treatment of UC, as well as providing compelling evidence for further research into the mechanism of BDMC in the treatment of UC.

## Figures and Tables

**Table 1:** Molecular docking of BDMC binding energy results for UC major targets.

Ligands	Receptors	PDB	Binding Affinity
BDMC	SRC	7NG7	−8.7 kcal/mol
BDMC	EGFR	1M14	−7.5 kcal/mol
BDMC	AKT1	7NH4	−9.2 kcal/mol
BDMC	PI3KR1	5M6U	−8.4 kcal/mol

## Data Availability

The data that endorse the results of this research are available upon reasonable request from the corresponding author (H.C).

## Supplementary Materials

The following supporting information can be downloaded at: https://www.mdpi.com/article/10.3390/molecules28010068/s1. Supplementary Table S1: The potential target genes of bisdemethoxycurcumin; Supplementary Table S2: The potential target genes of ulcerative colitis were retrieved from GeneCards; Supplementary Table S3: A total of 2601 ulcerative colitis target genes were obtained from five databases (DrugBank, GeneCards, OMIM, PharmGkb and TTD databases); Supplementary Table S4: The intersection of potential target genes of bisdemethoxycurcumin and ulcerative colitis.
